# The Nrf1 and Nrf2 Balance in Oxidative Stress Regulation and Androgen Signaling in Prostate Cancer Cells

**DOI:** 10.3390/cancers2021354

**Published:** 2010-06-21

**Authors:** Michelle A. Schultz, Asim B. Abdel-Mageed, Debasis Mondal

**Affiliations:** 1 Department of Pharmacology, Tulane University Medical Center, 1430 Tulane Avenue, New Orleans, LA 70112, USA; E-Mail: mschultz@tulane.edu; 2 Department of Urology, Tulane University Medical Center, 1430 Tulane Avenue, New Orleans, LA 70112, USA; E-Mail: amageed@tulane.edu

**Keywords:** Nrf1 (NF-E2 related factor-1), Nrf2 (NF-E2 related factor-2), prostate cancer, oxidative stress, androgen independence, androgen deprivation therapy, reactive oxygen species (ROS), androgen receptor (AR), di-hydrotestosterone (DHT)

## Abstract

Reactive oxygen species (ROS) signaling has recently sparked a surge of interest as being the molecular underpinning for cancer cell survival, but the precise mechanisms involved have not been completely elucidated. This review covers the possible roles of two ROS-induced transcription factors, Nrf1 and Nrf2, and the antioxidant proteins peroxiredoxin-1 (Prx-1) and Thioredoxin-1 (Txn-1) in modulating AR expression and signaling in aggressive prostate cancer (PCa) cells. In androgen independent (AI) C4-2B cells, in comparison to the parental androgen dependent (AD) LNCaP cells, we present evidence of high Nrf1 and Prx-1 expression and low Nrf2 expression in these aggressive PCa cells. Furthermore, in DHT treated C4-2B cells, increased expression of the p65 (active) isoform of Nrf1 correlated with enhanced AR transactivation. Our findings implicate a crucial balance of Nrf1 and Nrf2 signaling in regulating AR activity in AI-PCa cells. Here we will discuss how understanding the mechanisms by which oxidative stress may affect AR signaling may aid in developing novel therapies for AI-PCa.

## 1. Treatment of Castration Resistant Prostate Cancer: A Formidable Therapeutic Challenge

Prostate cancer (PCa) is the second leading cause of cancer related deaths in American males [1]. In the early stages of PCa, tumors are localized in the prostate and manifest androgen dependent growth. They are therefore more responsive to conventional treatments such as radical prostatectomy and hormonal therapy. Indeed, androgen deprivation therapy (ADT), designed to inhibit PCa growth through disruption of androgen receptor (AR) signaling, is successful during early stages of the disease [2]. However, the development of androgen independent (AI) or castration resistant PCa (CRPC) remains a significant obstacle to successful treatment because tumor cells become unresponsive to androgen deprivation and often metastasize to sequestered organs [3,4,5,6,7]. Chemotherapy can be used in these patients, but chemoresistance develops rapidly [8]. Novel therapeutic strategies to target the later stages of PCa will be of crucial importance. 

The aggressive nature of advanced PCa dictates morbidity and mortality. Several mechanisms have been suggested to be responsible for the development of androgen independent growth in PCa cells. Many of these pathways involve changes in the activity of various cofactors that modify AR signaling [9,10,11,12,13,14,15]. These cofactors can often enhance the sensitivity of AR to di-hydrotestosterone (DHT) or enable AR signaling in the absence of androgens. However, the precise mechanisms that control changes in AR activity during hormone fluctuations and ADT have not been elucidated [7,11,16]. It has been suggested that CRPC cells use residual levels of hormone (from adrenal and/or prostatic sources) to activate AR signaling during ADT [17,18]. The behavior of these cells is likely due to an adaptive response to a combination of stresses from various environmental factors that modify AR signaling in aggressive PCa cells. During advanced stages, metastatic cells often localize to areas of the body in which tumor hypoxia and changes in tumor vascularization may significantly affect reactive oxygen species (ROS) generation. Indeed, factors that modify oxidative stress signaling have recently been implicated in PCa aggressiveness and progression to androgen independence [7,13,14,19,20,21,22,23,24]. ADT can also induce oxidative stress through disruption of tumor vascularization [25,26,27,28]. 

## 2. NADPH Oxidases in Promoting Prostate Cancer Growth despite Hormonal Therapy

ROS (e.g., superoxide radicals, hydroxyl radical and peroxide radicals) act as potent second messengers in many signaling pathways. Numerous studies have demonstrated that cancer cells take advantage of downstream effectors of oxidative stress signaling [7,22,24,29]. ROS are known to regulate several physiological processes in normal cells, however, studies have also shown that in cancer cells, a differential regulation of ROS signaling can occur that may significantly affect tumorigenesis and aggressive growth [7,22,24,29,30]. In the tumor environment, there are several irregularities in the micro-circulation that cause intermittent blood flow. An unsteady supply of blood to cells results in poor oxygenation, thereby resulting in a state of hypoxia. Hypoxia has often been linked to the production of ROS [31,32]. It is likely that cells produce ROS in response to hypoxia in order to maintain sufficient levels of ROS for regulation of normal physiological processes. Hypoxia also plays a significant role in hormone signaling and has been shown to correlate with biochemical failure and poor clinical outcome [7,13,22,27]. Some have suggested that hypoxia increases sensitivity to DHT through selection of PCa cells with higher 5α-reductase activity, converting adrenal androgens into DHT, and by increasing the activity and sensitivity of AR [15,26,33]. Indeed, many of the factors that predispose individuals to prostate cancer (e.g., aging, obesity and hormonal fluctuations) are associated with progressively increased oxidative stress. Hence, delineation of the molecular pathways by which ROS enhance PCa aggressiveness could potentially aid in the development of new therapeutic strategies to target androgen independence.

**Figure 1:**
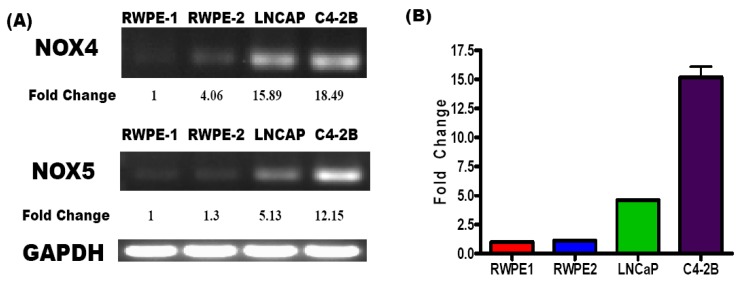
NOX4 and NOX5 mRNA Expression in a Prostate Cancer Progression Model. **(A)** RNA was isolated from PCa cell lines (RWPE1, RWPE2, LNCaP, and C4-2B) and NOX4 and NOX5 gene expression was evaluated by RT-PCR. GAPDH was used as an internal control. Fold changes in Nox4 and Nox5 mRNA expression were normalized to GAPDH. Numbers represent fold-changes as compared to normalized RWPE-1 expression (n = 3); **(B)** Quantitative RT-PCR (qRT-PCR) was done to evaluate NOX5 mRNA expression in RWPE1, RWPE2, LNCaP, and C4-2B cells (n = 2). GAPDH was used as the internal control.

Intracellular ROS can be generated through disruption of the mitochondrial electron transport chain or through activation of ROS producing enzymes like NADPH Oxidase [20,22,23,31,32,34]. NADPH oxidases (NOX) are a family of ROS producing enzymes that are traditionally associated with phagocytic cells but in recent years have been shown to be expressed in several non-phagocytic cell types. NOX enzymes convert oxygen into superoxide radicals, which are then converted into hydrogen peroxide (H_2_O_2_) by superoxide dismutase (SOD) [21,35]. Studies have demonstrated that H_2_O_2_ may be used as a second messenger in the regulation of several proliferation, differentiation, and growth factor signaling pathways in both normal and cancer cells. It has also been linked to enhanced survival and migration in cancer cells [21,36]. In pancreatic cancer cells, ROS production by NOX enzymes protects cells from apoptosis [29]. In these cells, the production of ROS by NOX4 was an integral part of apoptosis inhibition. High levels of ROS have also been shown to be constitutively produced in PCa cells and directly associated with an aggressive phenotype. Blocking NOX mediated ROS production with diphenyliodinum (DPI) has significant effects on cell proliferation and survival, whereas neutralization of ROS with N-acetyl cysteine is not as effective [24]. DPI also inhibited migration and invasion in aggressive PCa cell lines through inhibition of NOX1, a NOX involved in the regulation of angiogenesis. Also, siRNA to NOX5 suggested a significant role for NOX5 in ROS production in aggressive cells. Others have reported that inhibition of NOX5 induces apoptosis in PCa cells [23]. In aggressive PCa cell lines, several NOX isozymes (NOX2, NOX4, and NOX5) were shown to produce ROS [20]. In our studies using mRNA from RWPE1 and RWPE2 non-tumorigenic prostate cells and LNCaP (androgen dependent) and C4-2B (androgen independent) tumorigenic PCa cells, we observed that both NOX4 and NOX5 mRNA expression paralleled the aggressive nature of C4-2B cells (Figure 1). Furthermore, since NOX5 gene expression was expressed at the highest level in C4-2B cells, our findings implicate a clear relevance of the ROS induction machinery in progression of PCa cells towards a castration resistant phenotype.

Although androgens have been shown to significantly affect regulation of ROS expression, the associated mechanisms that regulate pro-oxidant and antioxidant enzyme expression and activity have not been completely elucidated. The above findings, which corroborate previously published data about aggressive PCa cells, implicated that increased NOX expression may generate a significant portion of the ROS required for aggressive cell growth [23]. The significant (p < 0.001) difference in NOX5 expression between LNCaP and C4-2B cells may also indicate a crucial role of ROS signaling mechanisms in regulating androgen independence. Indeed, some studies have suggested that the modification of NOX expression by androgen may have significant implications on PCa response to ADT [20,37,38,39]. Interestingly, in rat ventral prostate, the removal of androgens has been shown to induce ROS production, possibly via increased expression of NOX enzymes. However, replacement of androgen only partially reduced NOX expression in this study [20].

It is also possible that fluctuations in androgen levels, which might occur in the elderly patients with PCa, could gradually increase NOX levels because NOX expression is not completely restored to basal levels after androgen replacement. Based on the aforementioned notions, we postulate that castration induced NOX expression and the subsequent increases in ROS may stimulate mitogenic and survival pathways associated with increased ROS expression, especially during ADT. This would result in the selection and outgrowth of castration resistant tumor cell clones and increase the likelihood of aggressive PCa development [20]. Thus, a clearer understanding of the role of NOX-induced ROS and its downstream effectors in regulating PCa cell growth will be essential for discovery of novel therapeutic targets. 

## 3. Nrf1 and Nrf2 Transcription Factors: Master Regulators of Oxidative Stress Signaling

Excessive production of ROS can be detrimental to both normal and cancer cells. High levels of ROS damage lipids, DNA, and proteins in the cell, disrupting cell function. Therefore, advanced PCa cells may have developed mechanisms to utilize higher levels of ROS for mitogenic purposes by upregulating expression of antioxidant enzymes [24,40,41]. Numerous studies have investigated the role of antioxidant enzymes in cancer signaling and have seen that antioxidants have a significant role in regulation of cancer growth and survival [15,42,43,44]. Further understanding of the dysregulation of antioxidant signaling may give insight into the molecular mechanisms that regulate aggressive and androgen independent activity in PCa cells. 

Nrf1 (NF-E2 related factor-1) and Nrf2 (NF-E2 related factor-2) are two oxidative stress sensitive transcription factors that belong to the CNC/bZIP family of transcription factors consisting of NF-E2, Nrf1, Nrf2, Nrf3, BACH1, and BACH2 [45,46,47,48]. Both Nrf1 and Nrf2 are responsible for regulating the expression of many antioxidant genes including peroxiredoxin-1 (Prx-1), thioredoxin-1 (Txn-1), GCLC (Glutamate cysteine ligase catalytic subunit - an enzyme responsible for catalyzing the formation of glutathione), glutathione peroxidase (GPX-1), drug metabolizing enzymes (cytochrome P-450s), and several ATP Binding Cassette (ABC) transporters that are responsible for drug efflux [45,47,49,50]. All of these genes are essential to the maintenance of oxidative homeostasis and contain an Electrophile Response Element (EpRE) to which Nrf1 and Nrf2 bind (also known as the Antioxidant Response Element). Here we will use EpRE to describe this element to avoid confusion with the Androgen Response Element (ARE) that is regulated by the androgen receptor. 

Both Nrf1 and Nrf2 form obligate heterodimers with small Maf proteins like MafG. When activated by oxidative stress, these transcription factors interact with MafG to regulate the transcription of oxidative stress related genes [46,47,51,52,53]. Both Nrf1 and Nrf2 are essential to the cellular response to oxidative stress and several studies have shown that knockdown of Nrf1 and/or Nrf2 expression sensitizes cells to oxidative stress [54,55,56,57,58]. It has also been suggested that Nrf2 responds to inducible oxidative stimuli and that Nrf1 regulates more constitutive forms of oxidative stress [58]. Increased oxidative stress has been shown to promote tumor proliferation and survival through dysregulation of redox sensitive pathways [7,24,38,39,59,60].Here, we discuss the possible role of Nrf1 and Nrf2 in regulating the expression of antioxidant enzymes that can modulate AR signaling in PCa. 

**Figure 2:**
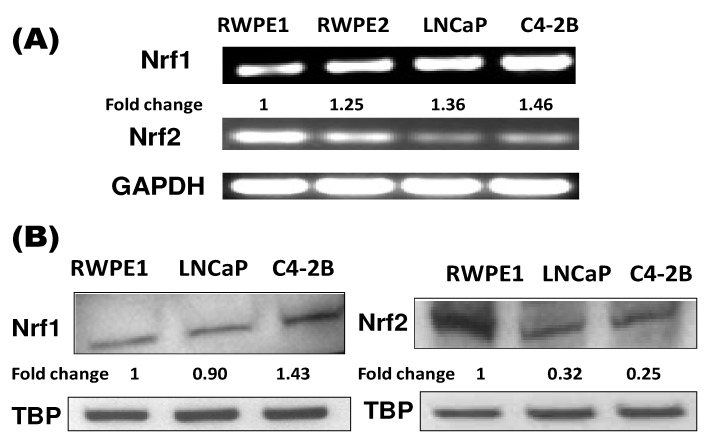
Nrf1 and Nrf2 Expression in Prostate Cancer Cell Lines. **(A)** RT-PCR for Nrf1 and Nrf2 mRNA expression in RWPE-1, RWPE-2, LNCaP, and C4-2B PCa cells are shown. GAPDH was used as the internal control (n = 3); **(B)** Nuclear Nrf1 and Nrf2 protein expression was measured in RWPE-1, LNCaP, and C4-2B PCa cells by western immunoblotting (n = 3). TATA Binding Protein (TBP) was used as internal control. Values are normalized to TBP and fold changes were calculated as compared to RWPE-1 cells.

We present data on the differential expression of Nrf1 and Nrf2 in androgen dependent and castration resistant PCa cell lines (Figure 2). Our data shows that Nrf1 is more highly expressed in the androgen independent C4-2B cells, as compared to their androgen dependent precursor, LNCaP cells. Also, in contrast to these two tumorigenic PCa cell lines, Nrf1 expression was lower and Nrf2 expression was significantly higher in the non-tumorigenic cell lines, RWPE1 and RWPE2. 

If Nrf1 is responsible for the regulation of constitutive forms of oxidative stimuli, the increased expression of Nrf1 suggests that C4-2B cells are more equipped for constitutive modification of antioxidant signaling, which could enhance the aggressive phenotype of these cells. Our findings indicate that suppression of Nrf2 and induction of Nrf1 may be responsible for modified antioxidant signaling in aggressive PCa cells. In addition to changes in NOX expression, this suggests that there is a differential regulation of ROS and antioxidant signaling in non-tumorigenic prostate cells and tumorigenic PCa cells.

## 4. Nrf2: Known Mediator of Mitogenic Signaling and Gene Expression in PCa Cells

Similar to our observations, Nrf2 expression has been shown to be decreased in human aggressive PCa tissues. This study demonstrated that inhibition of Nrf2 in PCa cells can result in higher levels of oxidative stress, resulting in increased DNA damage [60]. ROS induced DNA damage, as a result of decreased Nrf2 expression, might also enhance tumorigenicity by impairing a cell’s defense mechanisms during inflammation. It has been suggested that inflammation may be induced by fluctuations in hormone levels [61]. The severity of inflammation has also been shown to be regulated by Nrf2 [61]. Also, because an increased capacity to produce ROS is a characteristic of aggressive PCa, decreased Nrf2 expression may result in a higher steady state level of oxidative stress within the cell. It is likely that alternative methods of ROS management will be needed in these cells to prevent ROS induced apoptosis. 

Interestingly, deletion of Nrf2 in mice increases nuclear factor-kappaB (NF-κB) signaling [62]. This has significant implications on CRPC because NF-κB signaling is involved in PCa progression to androgen independence [63]. The expression of key Nrf2 regulated antioxidant enzymes (e.g., Glutathione-S-Transferases) also decreases during PCa progression [60]. To better understand the role that Nrf2 downregulation plays in PCa, it will be important to elucidate the mechanisms by which Nrf2 expression is decreased in aggressive PCa cells.

## 5. The Role of Nrf1 p65 and p120 Isoforms in Regulating Nrf2 Activity in PCa Cells

Nrf2 can be activated by ROS, reactive nitrogen species, heavy metals, and electrophilic xenobiotics. In its inactive state, Nrf2 is bound to its cytoplasmic chaperone, Keap1 [47,64,65]. Keap1 serves as a linker protein for Cul3 based E3 ubiquitination of Nrf2, which leads to Nrf2’s proteosomal degradation. When cells are exposed to one of a variety of stressors or Nrf2 inducing agents, Nrf2 dissociates from Keap1 and translocates to the nucleus to regulate transcription of EpRE containing genes. However, it has been suggested that Nrf2 dissociation from Keap1 may not be the only mechanism by which Nrf2 can be activated [66]. 

In contrast, Nrf1 in its native form is an endoplasmic reticulum resident protein with its N-terminal domain (NTD) integrated into the endoplasmic reticulum membrane and the remainder of the Nrf1 protein residing on the cytoplasmic side of the endoplasmic reticulum [48,67,68,69,70]. Interestingly, others have also shown that the full length p120 isoform of Nrf1 might also be found on the nuclear membrane and that several shorter isoforms of Nrf1 also exist (p36, p55, p65 and p95) [48,68]. The precise mechanism by which Nrf1 is broken down from its larger p120 isoform into its smaller active isoforms is unknown. 

Recent evidence suggests that Nrf1 is involved in regulation of the 26S proteosome’s transcription [71]. In response to proteosome inhibition by drug treatment, Nrf1 activates proteosome subunit transcription. We hypothesize that a possible mechanism for the processing of Nrf1 into its active isoforms is through Nrf1’s proteosomal degradation/processing, especially since Nrf1 regulates transcription of the catalytic subunit genes of the 26S proteosome. The 26S proteosome has been shown to activate NF-κB by processing it from its larger p105 isoform into its DNA binding p50 isoform [72,73]. It is well known that in order to regulate transcription, the inactive p120 isoform of Nrf1 must be broken down into its active smaller isoforms that do not contain the inhibitory NTD of the protein [48,67,68,69,70]. Keap1 has been shown to mediate Nrf2’s proteosomal degradation by facilitating the ubiquitination of Nrf2’s Neh2 domain [64,74]. For a proteosome to degrade a protein, it should be ubiquitinated [75]. Nrf1 contains sub-domains in its N-terminal acidic rich region that are similar to Nrf2’s Neh2 domain, which suggests that Nrf1 may have the ability to be ubiqitinated [69]. Therefore, it is quite possible that proteosomal processing of Nrf1 could remove the NTD and process Nrf1 into its smaller active isoforms. This hypothesis is further strengthened by Nrf1’s role in activating transcription of proteosome subunit genes in response to proteosome inhibition [71]. Without processing, Nrf1 would not be able to regulate transcription. This implies that an Nrf1 mediated feed-forward mechanism may regulate transcription of proteosome subunit genes in order to maintain the expression of active Nrf1 isoforms. If our hypothesis is true, inhibition of the proteosome would also inhibit activation of Nrf1 by inhibiting the processing of Nrf1 into its active isoform. To investigate the possible role of Nrf1 isoforms in LNCaP and C4-2B cells, we measured the basal expression of the p65 and p120 isoforms in nuclear extracts from these two PCa cell lines.

**Figure 3:**
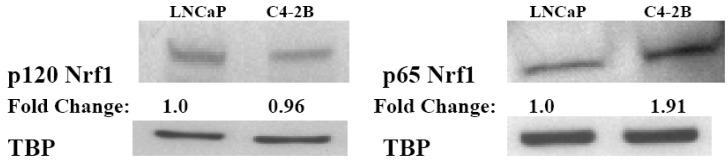
Expression of the p120 and p65 Isoforms of Nrf1 in PCa Cells. Westerns were carried out to measure levels of the p120 and p65 isoforms of Nrf1 in nuclear extracts from LNCaP and C4-2B cells. TBP was used as an internal control and Nrf1 protein levels were normalized to TBP and fold changes were calculated as compared to LNCaP cells (n = 2).

In our western immunoblot data (Figure 3), nuclear extracts of LNCaP and C4-2B cells showed both a p120 isoform and a p65 isoform of Nrf1. This suggests that the p120 isoform of Nrf1 could be located on the nuclear membrane of LNCaP and C4-2B PCa cells because the NTD that is present in the p120 isoform of Nrf1 anchors it to membranes. This implies that a mechanism of Nrf1 activation could be detachment from its NTD in the nuclear membrane followed by nuclear translocation and gene regulation. Due to the close proximity of the nuclear membrane to the ER, it is also possible for Nrf1 to relocate to the nuclear membrane in response to specific stimuli. The positioning of Nrf1 in the nuclear membrane may allow easy access to genes during the oxidative stress response, whereas Nrf2 must be translocated to the nucleus upon activation. Therefore, the increased expression and/or localization of Nrf1 to the nucleus in aggressive PCa cells, suggests that mechanisms of Nrf1 activation may be different in CRPC cells as compared to androgen dependent cells.

## 6. Nrf1 and Nrf2 Balance in Differential Regulation of Gene Expression via the EpRE

The combined activities of Nrf1 and Nrf2 protect cells during cellular stresses and may enable both normal and cancer cells to survive in toxic environments. Both Nrf1 and Nrf2 have the ability to regulate expression of antioxidant enzymes through their EpREs. There are some genes, such as the antioxidant proteins Prx-1 and Txn-1, whose transcription is regulated by both Nrf1 and Nrf2, depending on whichever transcription factor is available at the time and the tissue specific expression of Nrf1 and Nrf2. There are also others like NQO-1, and Metallothionein 1 and Metallothionein 2 (MT1 and MT2), that are preferentially regulated by either Nrf1 or Nrf2 [51,52,58]. The differential regulation of these genes may have significant implications on PCa aggressiveness and androgen independence. Several genes are known to be regulated by Nrf2, including electrophile conjugating enzymes, antioxidant proteins, and glutathione regulation enzymes, but less is known about Nrf1 specific regulation of EpRE regulated genes [45,46,47,49,50].

Ohtsuji *et al.* [58] demonstrated the distinct roles of Nrf1 and Nrf2 in activating EpRE regulated genes. This investigation showed that liver specific knockout of Nrf1 (total knockout kills mice by embryonic day 13) significantly downregulates expression of several genes that contain EpREs in their promoter. These include MT1 and MT2, GADD45γ, ATP Binding cassette sub family F (GCN20) member 1, and several other genes that have cell growth, signal transduction, transport, and glycosylation related functions. Conversely, Nrf2 had no effect on induction of MT1 and MT2 in mouse liver cells. Interestingly, MT1 and MT2 are expressed in PCa cell lines and have been shown to correlate strongly with Gleason score in patient samples [76,77,78]. In addition, MT expression has been shown to increase in response to hypoxia in PCa cells [76]. Ohtsuji *et al.* proposed that Nrf2 is crucial for survival during severe stress but that Nrf1 is indispensible for steady state stress under normal conditions. This suggests that there is indeed a specific role for Nrf1 in the regulation of EpRE genes. It also implies that cancer cells might be able to use Nrf1 to control the steady state levels of oxidative stress by continually altering levels of antioxidants and EpRE regulated enzymes in the cell.

Wang *et al.* [48] demonstrated that the p65 isoform of Nrf1 functions as a repressor of Nrf2 mediated gene regulation. Untreated cells were unaffected by changes in Nrf1 or Nrf2, but cells overexpressing p65 Nrf1 were more susceptible to H_2_O_2_ induced cell death, suggesting that this isoform can increase ROS expression through inhibition of Nrf2 activity. NQO-1 and GCLC, two EpRE containing antioxidant enzymes, were shown to be negatively regulated by overexpression of the p65 isoform of Nrf1. To regulate transcription of EpRE mediated genes, both Nrf1 and Nrf2 must first dimerize with small Maf proteins like MafG or MafK [47,52,65,79,80]. In this study, electrophoretic mobility shift assays (EMSAs), immunoprecipitation, and chromosomal immunoprecipitation (ChIP) assays revealed that Nrf1 has a greater capacity to bind with MafG than Nrf2. Nrf1 was also shown to more strongly bind to the EpRE of some genes than Nrf2 and repress Nrf2’s activity [48]. It is interesting to note that in our model of PCa progression, Nrf2 expression was lowest in C4-2B cells, which had the highest levels of Nrf1 expression. While it has been shown that Nrf1 overexpression decreases Nrf2 mediated gene activation, it has not been shown that Nrf1 affects the expression of Nrf2. Although further investigation will be needed, we propose that another mechanism by which Nrf1 represses Nrf2 function is through reduction of Nrf2 expression in PCa cells. We hypothesize that cancer cells modify the balance of Nrf1 and Nrf2 signaling and expression to create a favorable environment in which oxidative stress, due to changes in antioxidant expression, can continually be used to enhance cell growth. 

**Figure 4:**
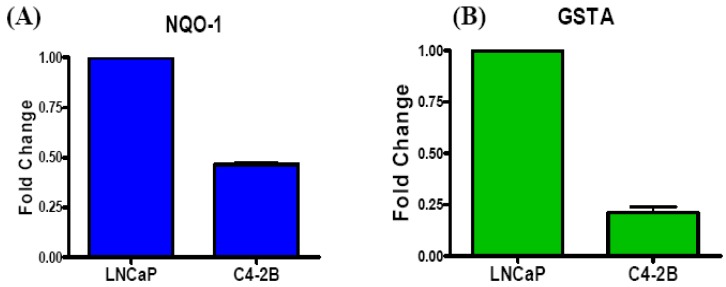
Differential Transcriptional Activation through the EpRE. In LNCaP and C4-2B cells. **(A)** an NQO-1 Luciferase reporter plasmid was transfected and **(B)** a GSTA Luciferase reporter plasmid (containing 6 repeats of the GSTA EpRE sequence) was transfected. After 24 h, cell extracts were obtained *luc*-activities were measured in a Dual Luciferase assay (Promega). A Renilla based reporter plasmid was used as the internal control (n = 2).

In the following figure, NQO-1 and GSTA luciferase assays were done to assess the differences between EpRE gene regulation by Nrf1 and/or Nrf2 in LNCaP and C4-2B cells. The NQO-1 gene, as previously mentioned, can be negatively regulated by Nrf1 overexpression and Glutathione-S-Transferase (GST) expression is diminished in aggressive Nrf2 deficient PCa tissues [48,60]. Hence, in our preliminary studies, we have utilized luciferase reporter plasmids containing NQO-1 and GSTA promoter sequences to evaluate differences in EpRE gene regulation by Nrf1 and/or Nrf2 in androgen dependent and castration resistant PCa cells (Figure 4). 

Our data show that in C4-2B cells, which have higher Nrf1 expression and lower Nrf2 expression than LNCaP cells, significantly lower transcriptional activation (luc-activity) was observed in the NQO-1 and GSTA promoters containing the EpRE. This corroborates our Nrf1 and Nrf2 expression data in C4-2B cells and implicates a direct role for increased Nrf1 activity and decreased Nrf2 activity in regulation of EpRE mediated gene transcription in the CRPC cells. In our proposed model of PCa, Nrf1 is overexpressed and Nrf2 is downregulated in aggressive CRPC cells. This results in disruption of the endogenous balance of EpRE mediated gene expression because not only is there more activation of Nrf1 specific genes, there is also decreased activation of Nrf2 specific genes due to Nrf1 mediated inhibition of Nrf2 activity. Expression of genes like Prx-1 and Txn-1 that can be regulated by both Nrf1 and Nrf2 will likely be maintained or enhanced in Nrf1 overexpressing cells, but levels of their expression would depend on the manner in which that gene is regulated by Nrf1 [58]. 

## 7. Prx-1 and Txn-1 Mediated Augmentation of AR Signaling Cascade in PCa Cells

Prx-1 is a remarkable thiol based peroxidase that can modify transcription factor activity [15,33,81]. In normal circumstances, it uses the peroxide reactivity of its cysteine sulfur atom to reduce hydrogen peroxide and organic peroxides and uses Txn-1 as its hydrogen donor for disulfide reduction of Prx-1 to return to its native state. Thioredoxin reductase transfers reducing equivalents from NADPH to Txn-1 to restore Txn-1 to its active state after reduction of Prx-1 [82,83,84,85]. Interestingly, in response to hypoxia/reoxygenation, cells overexpressing Prx-1 have a greater capacity to directly interact with AR to enhance AR’s transactivation [15]. Recent studies have also shown that Prx-1 overexpressing PCa cells are more sensitive to androgen stimulation [33]. In addition, it has been reported that Prx-1 can regulate activation of NF-κB [81]. The DNA binding domain (DBD) of nuclear NF-κB is inactive when oxidized. It was reported that nuclear Prx-1 did not affect NF-κB nuclear translocation, but enhanced NF-κB reporter activity, suggesting that nuclear Prx-1 may enhance NF-κB activity by reducing its DBD. Investigators also showed that peroxide tone in subcellular compartments is differentially controlled at the microcompartment level, suggesting a role for antioxidants in regulating compartmental oxidative stress and activating transcription factors. Under conditions of elevated oxidative stress, members of the peroxiredoxin family can become hyperperoxidized [86]. In this hyperperoxidized state, these Prxs display approximately four fold higher chaperone activity [87,88]. Prx-1 also has chaperone functions during hypoxia in PCa cells [15]. While Prx-1’s antioxidant function may not affect Prx-1’s chaperone activity in PCa cells, Prx-1’s state of peroxidation may change in response to hypoxia, enhancing Prx-1’s chaperone activity. This correlates with previous reports that demonstrated Prx-1’s role in enhancing AR transactivation. 

Txn-1 signaling has also been shown to enhance NF-κB signaling, likely through a mechanism similar to Prx-1 [89]. Since Txn-1 translocates to the nucleus in response to oxidative stress and is directly involved in Prx-1 regulation, both Txn-1 and Prx-1 could possibly work together to maintain compartmental ROS expression [81,89]. This has significant implications on the regulation of oxidative stress and NF-κB signaling in PCa. Because NF-κB signaling is important in PCa cell survival, an augmented capacity to use antioxidant proteins like Txn-1 and Prx-1 to activate transcription factors might also increase a cell’s defense against the induction of apoptosis. This implies that cells that have elevated levels of functional antioxidants, especially in the nucleus, might be able to survive higher levels of oxidative stress in addition to stimulating transcription through transcription factor activation.

Interestingly, in breast cancer cells, Txn-1 has been shown to differentially regulate estrogen receptor (ER) signaling [90]. Txn-1 binds to and reduces zinc fingers on proteins in the ER transcriptional complex to modify ER signaling in breast cancer cells. Txn-1 was also overexpressed in invasive breast cancer tissues. Another investigation revealed that overexpression of Txn-1 and other Nrf2 regulated genes enabled breast cancer cells to become resistant to tamoxifen, a drug used for hormonal therapy in breast cancer [45]. As that ER and AR have many similar functions and mechanisms, it is also possible that Txn-1 may modulate AR signaling in a similar fashion. Further investigations will be required to understand the role of Txn-1 in AR signaling and antiandrogen resistance. 

**Figure 5:**
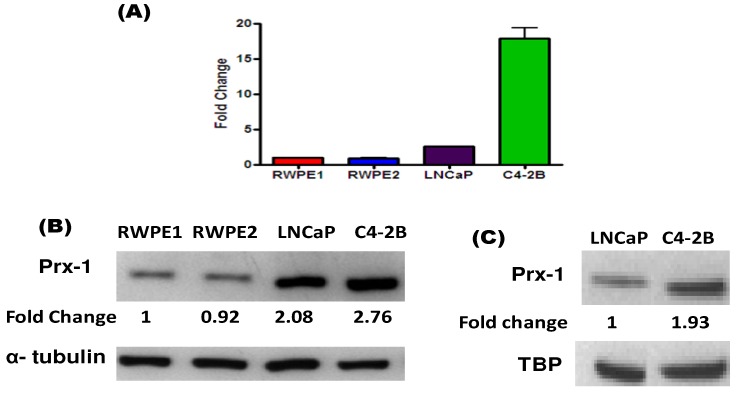
Prx-1 Expression in a Model of PCa Progression. **(A)** qRT-PCR and (**B)** total lysate westerns for Prx-1 in our panel of PCa progression. Data are normalized to GAPDH and α- tubulin respectively. **(C)** Nuclear Prx-1 protein levels in LNCaP and C4-2B cells are shown. Data are normalized to nuclear TBP levels.

In our preliminary studies, we also wanted to document whether the levels of Prx-1 in the PCa cells correlate with Nrf1 expression patterns in aggressive PCa cells. Similar to previous studies, we have measured Prx-1 gene expression in the four different PCa cell lines. In addition, we have carried out westerns to measure both cytosolic and nuclear Prx-1 levels in these cells (Figure 5). We have observed that in C4-2B cells, both Prx-1 mRNA and total protein expressions were higher than in RWPE-1, RWPE-2, LNCaP cells (Figure 5A and 5B). In addition, nuclear Prx-1 expression is almost 95% higher in C4-2B cells than in LNCaP cells (Figure 5C). Here we provide evidence that suggests that the Nrf1 and Nrf2 transcription factors may regulate the expression of antioxidant proteins that modulate hormone receptor signaling in cancer cells. Indeed, our current findings corroborate data from previous reports [15]. 

**Figure 6:**
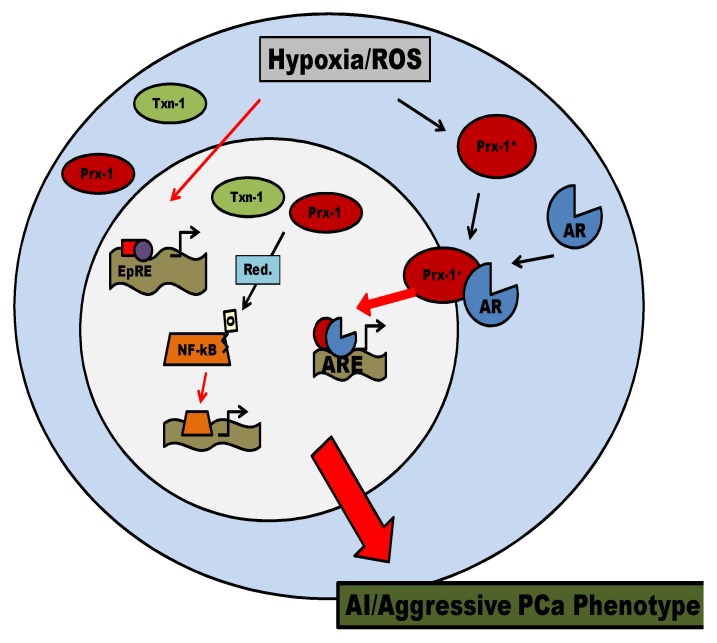
A Putative Model for Prx-1 and Txn-1 Mediated Signaling in PCa. In PCa cells, hormones, growth factors, inflammatory cytokines, and hypoxia can increase ROS levels, triggering Prx-1 chaperone activity and Txn-1 nuclear localization. The increased chaperone activity of Prx-1 allows enhancement of AR transactivation function in the nucleus. In addition, functional nuclear Prx-1 and Txn-1 may also activate NF-κB transactivation function via reducing its DNA binding domain [82,83,91]. Such a model may facilitate our understanding of how tumor growth may occur under circumstances of high oxidative stress leading towards an aggressive and androgen independent (AI) phenotype in PCa cell.

Collectively, the ability of Prx-1 to chaperone in elevated oxidative stress conditions and enhance AR transactivation combined with Prx-1 and Txn-1’s role in differentially regulating transcription factor activity suggests that aggressive PCa cells might use antioxidants to facilitate AR mediated signaling and growth during hypoxia and conditions of high oxidative stress. From recent findings on these two antioxidant proteins and their role in regulating activities of transcription factors, we proposed a model for their putative effects in cancer cells (Figure 6).

## 8. EpRE-Mediated Gene Expression in Androgen Independent PCa

It has been suggested that androgen ablation can lead to a state of hypoxia in PCa. As previously mentioned, MT, a gene whose expression has been shown to increase in response to hypoxia in PCa cells, is an Nrf1 regulated EpRE gene [58,76]. It was shown that human PCa tissues stain strongly for MT in residual cancer cells that had been exposed to androgen ablation. Collectively, this implies that Nrf1 overexpression in CPRC tissues could be responsible for the increased expression of MTs and other Nrf1 specific EpRE regulated genes in PCa tissues. More importantly, it also suggests that Nrf1 might be upregulated by hypoxia (and therefore ADT) in PCa cells. Further investigation must be carried out to delineate the role of Nrf1 in the response to hypoxia and ADT in prostate cancer. 

**Figure 7:**
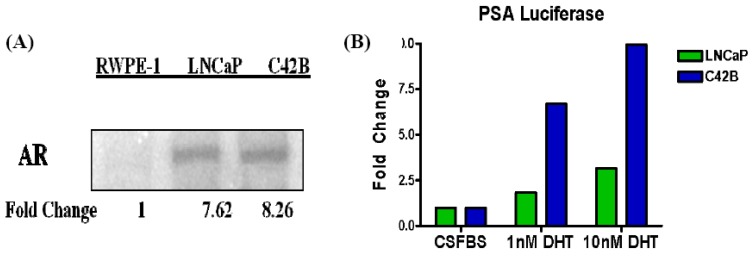
Differential regulation of AR-directed transcription in DHT stimulated cells. Lipofectamine was used to transfect the PSA Luc reporter plasmid into LNCaP and C4-2B cells. After transfection, cells were cultured in charcoal stripped FBS (CSFBS), either in the absence of DHT or in the presence of 1 nM or 10 nM DHT. Protein was harvested after 24 h of DHT stimulation then luciferase assays were carried out. Renilla was used as the internal control.

In order to correlate changes in antioxidant expression and signaling with increased AR signaling in aggressive PCa cells, we have carried out studies to monitor AR expression and AR-dependent transcription from the prostate specific antigen (PSA) promoter in LNCaP and C4-2B cells (Figure 7). In (A), Western immunoblot studies showed undetectable levels of AR expression in nontumorigenic RWPE-1 cells, and much higher AR expression in the tumorigenic LNCaP and C4-2B cells. However, although similar levels of AR expression were observed in both LNCaP and C4-2B cells, transient transfection studies using an AR dependent reporter plasmid (PSA-Luc) showed significant differences in AR-mediated activation (basal and DHT-induced) of the PSA promoter (B). As previously mentioned, CRPC cells (C4-2B) are more sensitive to hormone than androgen dependent (LNCaP) PCa cells. Our data supports this notion because castration resistant C4-2B cells were able to more strongly activate reporter activity even at low concentrations of DHT (1 nM). 

**Figure 8:**
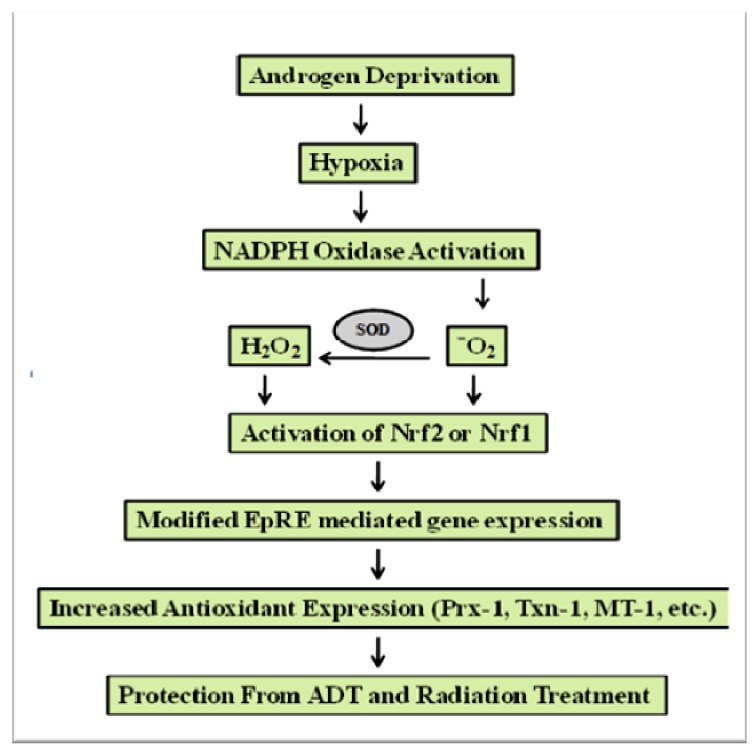
An Integrated Model for Nrf1/Nrf2 Balance in Regulating EpRE-Mediated Gene Expression in Androgen Independent PCa Cells. Androgen deprivation, through disruption of tumor vasculature, enables a state of hypoxia [7]. Hypoxia activates NOX activity, which in turn increases oxidative stress through increased production of ^-^O_2_,which is converted into H_2_O_2_ by superoxide dismutases [21,34]. Both O_2_^-^ and H_2_O_2_ can activate Nrf1 or Nrf2 signaling, which results in increased EpRE mediated gene expression (Prx-1, Txn-1, *etc*.) [49,56,79,92]. With upregulation of these antioxidant enzymes, aggressive cancer cells can protect themselves from ADT and radiation treatment [50,85,93,94]. This correlates with increased activation of AR regulated genes in aggressive PCa cells.

The above findings on differential AR signaling in LNCaP and C4-2B cells directly correlate with differences in expression of the oxidative stress related factors, Nrf1, Nrf2, Prx-1, Txn-1, Nox-4, and Nox-5. These findings strengthen our hypothesis that oxidative stress and a balance between Nrf1 and Nrf2 may regulate androgen signaling in PCa cells. Since AR mediated signaling is known to be regulated via hypoxia and ROS in PCa cells, it is probable that C4-2B cells have an increased capacity to maintain higher ROS levels (possibly via NOX), which results in increased expression of antioxidant proteins that can enhance AR signaling. It is possible that in aggressive PCa cells, Nrf1 overrides Nrf2 mediated regulation of EpRE genes because Nrf1 has a greater affinity to bind the EpREs of some antioxidant genes, especially if Nrf1 expression is higher than Nrf2 [48]. Due to the ability of Nrf1 to regulate many of the same genes as Nrf2, it is also possible that cells with diminished Nrf2 levels use Nrf1 as a backup for maintenance of antioxidant expression [58]. Therefore, in the following figure (Figure 8), we present a model to describe the role of oxidative stress in regulating the aggressive behavior of PCa cells.

## 9. Implications for Nrf1/Nrf2 in AR Expression: EpRE and TCF11/MafG Binding Sites

AR is responsible for differentiation of male sex characteristics and Nrf1 has been shown to be involved in bone differentiation [95,96,97]. AR is overexpressed in many PCa cells and is essential for survival of most PCa cells. Indeed, several studies have shown that one of the mechanisms by which castration resistant cells can adapt to low hormone conditions during ADT is by increasing the amount of AR present in the cell [98,99]. As previously discussed, oxidative stress has also been shown to modulate AR signaling in PCa cells and hypoxia may occur as result of ADT mediated changes in tumor vascularization. Therefore, because ADT may induce changes in AR expression, it is possible that oxidative stress regulated factors regulate AR expression in cells whose antioxidant system has been modified. 

**Figure 9:**
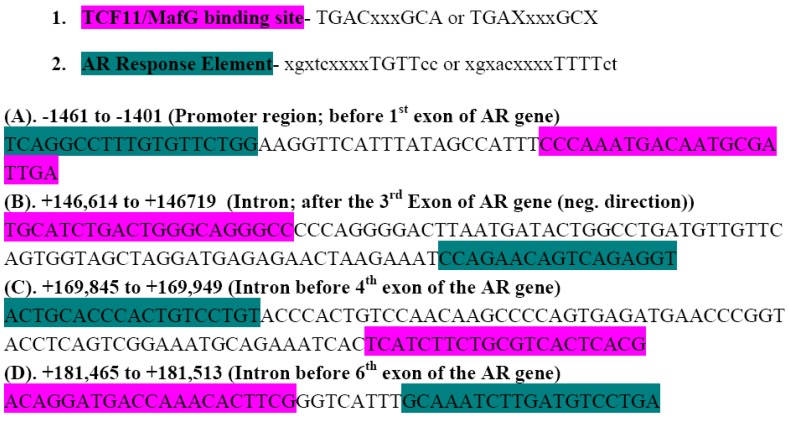
Possible Nrf1 and AR Binding Sites in the AR gene. The Genomatix MatInspector was used to search for transcription factor binding sites within the AR gene. Consensus sequences for the TCF11/MafG (pink) and ARE (teal) are provided at below.

In addition to the EpRE, Nrf1 can bind to a sequence called TCF11/MafG. Traditionally, TCF11 is the name for the full length Nrf1 isoform, but in this case, the TCF11 binding site refers to a site that could be bound by any shorter length Nrf1 isoform (p95 to p36) that does not contain the N-terminal domain (NTD) of Nrf1. The NTD of Nrf1 is inhibitory and sequesters Nrf1 in the endoplasmic reticulum (or possibly nuclear) membrane of a cell. In the human AR gene sequence, there are many sites that contain the TCF11/MafG binding sequence. Using the Genomatix MatInspector program (Ann Arbor, MI, USA), we searched the preceding 2000 bp before each exon in the human AR gene sequence for EpRE/Maf, TCF11/MafG, or ARE (Androgen Response Element) binding sites. We then mapped out AR and TCF11/MafG binding sites and observed several areas within the AR gene in which these TCF11/MafG and ARE sequences were in close enough proximity to each other to suggest an interaction between AR and Nrf1 (Figure 9). 

Interestingly, in the intron between the fifth and sixth exon of the AR gene, there is a sequence in which there is a TCF11/MafG binding site within 8 bp of an ARE binding site. This implies that under as yet unknown conditions, interactions between Nrf1/MafG and AR might occur to regulate AR transcription. The ability of both Nrf1 and AR to respond to oxidative stress signaling and to regulate differentiation processes suggests that Nrf1 and AR might also be able to interact during differentiation in normal development or possibly during cancer progression [100]. Because we have seen that Nrf1 is more highly expressed in aggressive PCa cells, we hypothesize that aggressive cells utilize interactions between Nrf1 and AR to modify AR mediated transcription. We believe that PCa cells might also use this mechanism to progress to an androgen independent state because an Nrf1 and AR mediated upregulation of AR transcription in PCa cells could be maintained by oxidative stress, rather than DHT, which would otherwise be needed to activate AR transcription. 

The 26S proteosome is responsible for degradation of ubiquitinated proteins, which helps maintain intracellular protein homeostasis. In a recent publication, it was shown that Nrf1 and not Nrf2 was necessary for the induction of 26S proteosome subunit genes after proteosome inhibition [71]. The induction was mediated through an EpRE in the promoter of the proteosome subunit genes. In LNCaP cells, Nrf1 was shown to be essential for activation of the EpRE of 26S proteosome subunit genes after drug induced proteosome inhibition [71]. Mutation of the EpRE in a luciferase construct of the subunit gene drastically inhibited gene activation. Interestingly, the 26S proteosome has also been shown to be involved in the regulation of AR expression. Suppression of the 26S proteosome activity with proteosome inhibitors downregulates AR expression. As previously mentioned, we hypothesize that the proteosome could play a role in processing Nrf1 into its active isoforms in a manner similar to NF-κB [72,73]. Given the possibility of Nrf1 regulating AR expression through activation of the above binding sites, increased expression of the active isoforms of Nrf1 could lead to increased activation of AR gene transcription. This hypothesis is further strengthened by the fact that inhibition of the proteosome leads to decreased AR expression in PCa cells [71]. Proteosome inhibition would be grounds for Nrf1 mediated induction of 26S proteosome subunit genes because inhibition of the proteosome would decrease the processing of Nrf1 into its active isoforms. 

## 10. Targeting of Nrf1/Nrf2 Mediated Changes in AR Signaling and Androgen Independence

To suppress the facilitative effects of ROS in PCa cells, numerous studies have used antioxidants (e.g., Selenium and Vitamin E) to quench ROS [1,101]. However, due to the complexity of antioxidant regulated pathways, attempting to inhibit antioxidant signaling through simple removal of ROS with antioxidant drugs may not be sufficient because a network of antioxidant proteins, transcription factors, and ROS producing enzymes work together to maintain the ideal oxidative environment within cancer cells [7,13,14,26,27,101,102,103,104]. Simple removal of ROS will activate a feedback response in cells (possibly via NOX) and ROS levels will be restored (e.g., NOX activation by hypoxia). 

A recent study showed that LAPC-4 prostate cancer cells have low expression of Nrf2 protein [8]. Another investigation showed that in LAPC-4 cells, the antioxidant drug genistein protects PCa cells from oxidative stress related DNA damage [105]. Genistein has also been shown to induce Nrf1 activity [106]. If patterns of Nrf1 and Nrf2 expression in LAPC-4 and other PCa cells change in a fashion similar to LNCaP and C4-2B cells, Nrf1 activity might also be induced in LAPC-4 cells in response to genistein. Although this study did not monitor Nrf1 or Nrf2 expression patterns in response to genistein, exposure to genistein inhibits LNCaP but not C4-2 (a C4-2B precursor) cell growth [107]. This further implicates the role of differential Nrf1 signaling in androgen dependent PCa cells and CPRC cells. Altogether, this is a classic example of why antioxidant drugs may work well in cancer prevention, but are often ineffective in aggressive cancer cells that already have altered antioxidant and pro-oxidant expression. After a cancer cell has adapted and modulated the antioxidant system to promote acceleration of cell growth, removal of the “ligand” (ROS) will likely only be able to temporarily inhibit antioxidant signaling in cancer cells. We suggest that the key to effectively using antioxidant drugs for cancer treatment is to find drugs that will target the signaling pathways associated with ROS and not ROS itself.

There are also other natural products that have been shown to reduce ROS in normal cells and increase ROS in cancer cells [108]. These types of drugs might be more efficacious for patients that already have cancer if they can target some of the mechanisms/proteins that regulate ROS production and neutralization in cancer cells. The advantage to using these drugs is that they are often less toxic and can be used to reduce baseline ROS levels. Long term ingestion of antioxidant drugs and natural products is thought to be effective in preventing the development of cancer, presumably through inhibition of DNA damage from elevated ROS or by lowering baseline ROS expression [101]. Indeed, the use of these agents in a preventative capacity is likely to provide the most benefit. We have recently published an article entailing the mechanism by which thymoquinone (TQ), a drug derived from black seed oil, can induce ROS, inhibit cell growth, and induce apoptosis in aggressive PCa cells but not in non-tumorigenic prostate cells [109]. TQ induced expression of apoptosis related enzymes and downregulated expression of prosurvival enzymes. Further investigation will have to be done to determine the usefulness of drugs like TQ in inhibition of PCa cell growth. 

Lastly, a more direct inhibition of Nrf1, Txn-1, or Prx-1 expression could be a possible mechanism for disruption of PCa cell growth. The utility of short interfering RNA (siRNA) or micro-RNA (miRNA) strategies to specifically suppress the expression of these genes in PCa cells may be an attractive candidate for anti-tumor gene therapy approaches in the future. In the meantime, the discovery of other drugs that can target antioxidant activity rather than ROS expression might be able to significantly improve PCa treatment strategies by inhibiting antioxidant mediated androgen independent activity. These types of drugs could be used as adjuvant therapy to highly toxic chemotherapy. Research on Nrf1 and Nrf2 regulation is relatively new, and not much is known about which specific drugs can negatively regulate Nrf1 or Nrf2 expression or activity. Several drugs have been shown to induce Nrf1 and Nrf2 activity, but further investigation into which drugs can inhibit their activity will have to be done in order to fully utilize Nrf1, Nrf2, and EpRE regulated genes as targets for CRPC [68,106,107]. 

## 11. Conclusions

Androgen deprivation therapy (ADT) is often used for the treatment of PCa patients. However, the development of CRPC remains a significant barrier to successful therapy. AR is functionally expressed in all stages of PCa, although CRPC cells often overexpress AR and employ alternative signaling cascades to enhance AR signaling. Elevated oxidative stress is associated with increased aggressiveness in PCa. Delineation of how oxidative stress and antioxidant signaling pathways are utilized by CRPC cells will implicate novel targets for abrogation of aggressive tumor cell growth in the later stages of PCa. We have used two sets of syngeneic PCa cell lines to emulate the natural progression of PCa *in vitro* from a non-tumorigenic stage (RWPE-1 and RWPE2 cells) to a tumorigenic and androgen dependent (LNCaP cells) and castration resistant (C4-2B cells) state. The growth advantage of CRPC cells during ADT may be manifested by a battery of both protective and signal enhancing antioxidant enzymes and transcription factors that enable these cells to maintain their aggressive nature and utilize AR signaling in the presence of low levels of androgen. In this review we have discussed several of the possible mechanisms by which PCa cells use Nrf1, Nrf2, and EpRE regulated genes to enhance aggressiveness and androgen responsiveness. 

We have provided new data showing that increased expression of the ROS producing enzymes NOX4, NOX5, and the antioxidant Prx-1 correlates with increased expression of Nrf1 in aggressive PCa cells. Nrf1, a dominant negative regulator of Nrf2 activity (and possibly expression), is expressed in both LNCaP and C4-2B cells, but is more highly expressed in C4-2B cells. The increased expression of nuclear Nrf1 and Prx-1 correlated with increased activation of the PSA promoter by AR in DHT treated C4-2B cells, as compared to LNCaP cells. We have discussed the role of the Nrf1 and Nrf2 transcription factors and role of the antioxidant enzymes Prx-1 and Txn-1, as mediators of hormone receptor signaling in cancer cells, possibly via their chaperone activities and/or transcription factor activation functions. We also present evidence that several regions within the AR gene contain EpRE binding sites that are proximal to the ARE, implicating an association between Nrf1 and AR at the DNA level for regulation of AR transcription. Lastly, we have discussed how increased EpRE regulated gene expression and activity, and a balance between Nrf1 and Nrf2 may ultimately regulate hormone receptor activity and androgen resistance in CRPC cells.
